# Simulation-based inference of the time-dependent reproduction number from temporally aggregated and under-reported disease incidence time series data

**DOI:** 10.1098/rsta.2024.0412

**Published:** 2025-04-02

**Authors:** Isaac Ogi-Gittins, Nicholas Steyn, Jonathan Polonsky, William S. Hart, Mory Keita, Steve Ahuka-Mundeke, Edward M. Hill, Robin N. Thompson

**Affiliations:** ^1^ Mathematics Institute, University of Warwick, Coventry, UK; ^2^ Zeeman Institute for Systems Biology and Infectious Disease Epidemiology Research (SBIDER), University of Warwick, Coventry, UK; ^3^ Department of Statistics, University of Oxford, Oxford, UK; ^4^ Geneva Centre of Humanitarian Studies, University of Geneva, Geneva, Switzerland; ^5^ Mathematical Institute, University of Oxford, Oxford, UK; ^6^ World Health Organization, Regional Office for Africa, Brazzaville, Republic of the Congo; ^7^ Faculty of Medicine, Institute of Global Health, University of Geneva, Geneva, Switzerland; ^8^ National Institute of Biomedical Research, Kinshasa, Democratic Republic of the Congo; ^9^ Civic Health Innovation Labs and Institute of Population Health, University of Liverpool, Liverpool, UK; ^10^ NIHR Health Protection Research Unit in Gastrointestinal Infections, University of Liverpool, Liverpool, UK

**Keywords:** infectious disease outbreak, time-dependent reproduction number, public health measures, approximate Bayesian computation, Ebola virus disease, stochastic simulations

## Abstract

During infectious disease outbreaks, the time-dependent reproduction number ( $R_{t}$ ) can be estimated to monitor pathogen transmission. In previous work, we developed a simulation-based method for estimating $R_{t}$ from temporally aggregated disease incidence data (e.g. weekly case reports). While that approach is straightforward to use, it assumes implicitly that all cases are reported and the computation can be slow when applied to large datasets. In this article, we extend our previous approach and develop a computationally efficient simulation-based method for estimating $R_{t}$ in real-time accounting for both temporal aggregation of incidence data and under-reporting (with a fixed reporting probability per case). Using simulated data, we show that failing to consider stochastic under-reporting can lead to inappropriately precise estimates, including scenarios in which the true $R_{t}$ value lies outside inferred credible intervals more often than expected. We then apply our approach to data from the 2018 to 2020 Ebola outbreak in the Democratic Republic of the Congo (DRC), again exploring the effects of case under-reporting. Finally, we show how our method can be extended to account for temporal variations in reporting. Given information about the level of case reporting, our framework can be used to estimate $R_{t}$ during future outbreaks with under-reported and temporally aggregated case data.

This article is part of the theme issue ‘Uncertainty quantification for healthcare and biological systems (Part 2)’.

## 1. Introduction

When an infectious disease outbreak is ongoing, estimates of pathogen transmissibility provide public health policy advisors with information about the progression of the outbreak and the effectiveness of control measures [1–4]. Alongside other quantities [5,6], a key metric is the time-dependent reproduction number ( $R_{t}$ ), which represents the expected number of secondary infections generated by a host who is infected at time t [7–11].

If the value of $R_{t}$ is (and remains) above one, then an outbreak will grow. If $R_{t}$ is below one instead, then an outbreak will decline. If $R_{t}$ is above one, then its value provides information about the proportion of transmissions that must be prevented for the outbreak to be brought under control. As well as being useful to inform interventions, estimates of $R_{t}$ are helpful for policy advisors to communicate changes in transmission to the public [12]. Accurate inference of $R_{t}$ and quantification of uncertainty in $R_{t}$ estimates are therefore essential.

A widely used approach for estimating $R_{t}$ (and quantifying associated uncertainty) was developed by Cori *et al*. [7]; we refer to that approach as the Cori method. Under the Cori method, $R_{t}$ is estimated from the disease incidence time series and the serial interval distribution (which characterizes the duration of the period between symptom onset times in infector–infectee transmission pairs). The Cori method has been popularized by the R software package *EpiEstim* [13], and is the basis for the online RShiny application *EpiEstim App* [14]. Multiple extensions to the Cori method exist, allowing explicit incorporation of features of real-world outbreaks such as imported cases [8,15], different pathogen variants [16] and heterogeneity in transmissibility between infected individuals [17,18].

Despite the substantial interest in estimating $R_{t}$ , until recently inference has been beset by temporal aggregation of disease incidence data [19,20]. This is a particular issue when the timescale of transmission is shorter than the timescale of data reporting. In the transmission model underlying the Cori method, an assumption is made that all cases arising at timestep t were generated by individuals who were themselves cases strictly prior to timestep t . In other words, if data are reported on a weekly basis and the Cori method is applied directly, then it is assumed that an infector and an infectee could not appear as cases in the same week. Since transmission of many pathogens can occur on the timescale of days, rather than weeks, this can lead to misspecification of the time between cases, biasing $R_{t}$ estimates. To address this, Nash *et al.* [21] developed a statistical framework for estimating $R_{t}$ from weekly incidence data, based on first inferring the expected daily incidence underlying the weekly reported values. Ogi-Gittins *et al.* [20] developed an alternative framework for estimating $R_{t}$ from temporally aggregated data (referred to here as the OG1 method), accounting for the full range of possible daily incidence time series that could underly the weekly case reports. The OG1 method involves repeatedly simulating a stochastic renewal equation model with a daily timestep (using different values of $R_{t}$ ) until a large number of simulations have been generated that exactly match the weekly incidence data. By generating simulations with a shorter timestep than that of the data, the issue described above is avoided. While the OG1 method allows $R_{t}$ to be estimated straightforwardly (the method simply requires repeated model simulation), a key drawback is that generating simulations that exactly match the observed data can require substantial computational resources, particularly for large outbreaks.

In addition to temporal aggregation of disease incidence data, another issue that limits the ability of policy advisors to gain an accurate picture of an unfolding outbreak is case under-reporting [22–24]. Here, we use this term to include both case under-ascertainment (i.e. infected individuals who do not attend healthcare services) and failures in the reporting system (i.e. individuals who attend healthcare services but are not recorded) [25]. It might be expected that under-reporting does not affect $R_{t}$ estimates, since, for example, 25 individuals infected at time t being expected to generate 50 secondary cases is equivalent in terms of $R_{t}$ to 100 infected individuals infected at time t being expected to generate 200 secondary cases (both scenarios correspond to $R_{t}=2$ ). However, owing to its stochastic nature, under-reporting generates uncertainty in the true disease incidence, with (as we show) the potential to affect $R_{t}$ estimates.

In this article, we extend the OG1 method and develop a simulation-based approach for estimating $R_{t}$ that accounts for both temporal aggregation of disease incidence time series and case under-reporting (based on an assumed probability that any given case is reported). We refer to our extended approach as the OG2 method. In contrast to the OG1 method, which requires stochastic model simulations to be generated that match the observed weekly incidence data exactly, in the OG2 method information about the value of $R_{t}$ is gained from any simulation in which the simulated number of cases in a given week is equal to or exceeds the number of reported cases in that week. This is because, in the presence of stochastic under-reporting, any simulation yielding more cases than the actual number of reported cases could be consistent with the disease incidence dataset.

We first use simulated datasets to show that the OG2 method can generate more accurate estimates of $R_{t}$ when applied to weekly aggregated and under-reported incidence data than naive application of the widely used Cori method (§3a). In that analysis, we apply the Cori method to weekly incidence data that are simply scaled up by a constant factor based on the case reporting probability (i.e. the number of cases each week is increased by a constant multiplicative factor, without accounting for the stochastic nature of reporting), whereas the OG2 method accounts for both temporal aggregation of the disease incidence time series and stochastic under-reporting. We also demonstrate that the OG2 method characterizes the uncertainty in $R_{t}$ estimates more appropriately than the OG1 method when the OG1 method is applied to the ‘scaled-up’ incidence data (§3a). We then use simulated datasets with large numbers of cases to demonstrate that the OG2 method is more computationally efficient than the OG1 method (§3b). We apply the OG2 method to data from the 2018 to 2020 Ebola outbreak in the Democratic Republic of the Congo (DRC), and show that accounting for stochasticity in under-reporting using the OG2 method leads to more appropriate credible intervals in $R_{t}$ estimates than applying the OG1 method to weekly incidence data that are again scaled up by a constant factor based on the case reporting probability (§3b). We use the Ebola outbreak dataset as the basis for an investigation into the effect of the reporting probability on $R_{t}$ estimates obtained using the OG2 method (§3c). Finally, we show how the OG2 method can be applied in more complex scenarios than those considered in our initial analyses, by considering simulated outbreak datasets in which the level of case reporting varies temporally (§3d). Our results indicate that careful assessment of the extent of under-reporting may be crucial for appropriate quantification of uncertainty in $R_{t}$ estimates during future infectious disease outbreaks.

## 2. Methods

Here, we describe the three methods for inferring $R_{t}$ that we used in our analyses and the datasets to which we applied those methods. First, we describe the naive application of the Cori method [7] to weekly incidence data, using a serial interval distribution that is discretized into weekly timesteps. Under that approach, neither temporal aggregation of the disease incidence time series nor under-reporting is accounted for. Second, we summarize the OG1 method [20], in which temporal aggregation of the incidence data is accounted for but under-reporting is not accounted for. Third, we describe our novel simulation-based approach (the OG2 method), that accounts for both temporal aggregation of the incidence data and under-reporting. Finally, we describe the serial interval distribution that we used in our analyses, and the simulated and real-world datasets that we used to test the performance of these approaches. We note that, while the Cori method and the OG1 method do not themselves account for under-reporting, in some of our analyses we apply those methods to incidence data that have been scaled up by a constant factor based on the case reporting probability. In those analyses, under-reporting is therefore accounted for in a naive fashion, neglecting stochasticity in reporting.

In the literature, it is common to attempt to infer $R_{t}$ from the incidence of cases and the serial interval distribution (as we do here when we apply each of the three inference methods). These quantities are more readily observed or estimated than the incidence of infections and the generation time distribution (the probability distribution characterizing the duration of the period between infection times in infector–infectee transmission pairs). Some methods for estimating the incidence of infections [26,27] and the generation time exist [28–30], but this is not the focus of the current study.

### (a) Cori method

Under the Cori method, the number of cases in any week t , $I_{t}$ , is assumed to be drawn from a Poisson distribution with mean


$$E\left(I_{t}|{\left\{I_{k}\right\}}_{k=1}^{t-1},R_{t},w\right)=R_{t}\sum_{s=1}^{t-1}w_{s}I_{t-s},$$

in which $w_{s}$ is the probability that the weekly discretized serial interval takes the value s weeks. The notation w denotes the set of values of $w_{s}$ ( s=1,2,3,… ).

Based on this transmission model, if the prior for $R_{t}$ is assumed to be a gamma distribution with shape parameter α and rate parameter β , then the posterior for $R_{t}$ is also a gamma distribution, with shape parameter $\alpha +I_{t}$ and rate parameter $\beta +\sum_{s=1}^{t-1}w_{s}I_{t-s}$ . A derivation of this result is provided in [20]. In our results, we considered the mean and central 95% credible interval of the posterior for $R_{t}$ . We note that, in the literature, when the Cori method is applied to daily disease incidence data (rather than weekly data as we considered here), it is common to incorporate a smoothing window to reduce the noise in $R_{t}$ estimates. Since we used weekly aggregated data that are less susceptible to day-to-day stochasticity than daily data, we did not apply a smoothing window here (or, equivalently, the posterior can be thought of as a simple application of the Cori method to weekly incidence data with a smoothing window of length one week).

When disease incidence is low, there may not be sufficient data to obtain robust $R_{t}$ estimates (see web appendix 3 of the article by Cori *et al.* [7] for further discussion about this; see also [31]). We therefore discarded $R_{t}$ estimates for which the width of the inferred 95% credible interval was larger than 75% of the width of the central 95% interval of the prior (i.e. the interval between the 2.5th percentile and 97.5th percentile values of the prior). We performed this same post-processing step for both the OG1 and OG2 methods.

### (b) OG1 method

The OG1 method provides a simulation-based approach for estimating $R_{t}$ , accounting for temporal aggregation of the disease incidence time series. When we used the OG1 method, we divided each weekly timestep into P=7 daily timesteps. The number of cases arising on the i th day of week t , denoted $I_{7\left(t-1\right)+i}^{\left(7\right)}$ (for i=1,2,…,7 ), was then assumed to be drawn from a Poisson distribution with mean


(2.1) $$E\left(I_{7\left(t-1\right)+i}^{\left(7\right)}|{\left\{I_{k}^{\left(7\right)}\right\}}_{k=1}^{7\left(t-1\right)+i-1},R_{t},w^{\left(7\right)}\right)=R_{t}\sum_{s=1}^{7\left(t-1\right)+i-1}w_{s}^{\left(7\right)}I_{7\left(t-1\right)+i-s}^{\left(7\right)},$$

in which $w_{s}^{\left(7\right)}$ is the probability that the daily discretized serial interval takes the value s days, and $w^{\left(7\right)}$ denotes the set of values of $w_{s}^{\left(7\right)}$ ( s=1,2,3,… ). This renewal equation is analogous to the one underlying the Cori method as described in §2a, but with a daily (rather than weekly) timestep.

The OG1 method involves repeated simulation of this renewal equation model using an iterative version of Approximate Bayesian Computation. First, the value of $R_{2}$ is estimated, followed by $R_{3}$ and so on.

To estimate $R_{2}$ , we ran simulations of the number of cases each day in week two. In each simulation, we: (i) sampled $R_{2}$ from the prior (i.e. from a gamma distribution with shape parameter α and rate parameter β ); (ii) distributed the reported cases in week one uniformly at random among the daily timesteps in week one; (iii) simulated the number of cases each day in week two. We repeated this procedure until we had generated M simulations in which the total number of simulated cases in week two matched the number of cases in the incidence dataset in week two. The mean estimate of $R_{2}$ and the corresponding 95% credible interval were then calculated from the values of $R_{2}$ used in the ‘matching’ simulations. For each matching simulation, the number of cases each day (i.e. ${\left\{I_{i}^{\left(7\right)}\right\}}_{i=1}^{14}$ ) was stored.

To estimate $R_{t}$ for t≥3 weeks, we ran simulations of the number of cases each day in week t . In each simulation, we: (i) sampled $R_{t}$ from the prior (i.e. from a gamma distribution with shape parameter α and rate parameter β ); (ii) sampled the daily incidence prior to week t from the matching sets stored when estimating $R_{t-1}$ ; (iii) simulated the number of cases each day in week t . We repeated this procedure until M simulations had been generated in which the total number of simulated cases in week t matched the number of cases in the incidence dataset in week t . Again, the mean estimate of $R_{t}$ and the corresponding 95% credible interval were then calculated from the values of $R_{t}$ underlying the matching simulations. For each matching simulation, the number of cases each day (i.e. ${\left\{I_{i}^{\left(7\right)}\right\}}_{i=1}^{7t}$ ) was stored.

### (c) OG2 method

Although the OG1 method is simple to apply, generating simulations that match the observed incidence data each week exactly can be time consuming, and under-reporting is not accounted for. We therefore modified the OG1 method, giving rise to the OG2 method that accounts for stochastic under-reporting (each case is reported with probability ρ ) and is computationally efficient to run.

In the OG2 method, unlike in the Cori method or OG1 method, we differentiated between the number of cases in week t ( $I_{t})$ and the number of reported cases in week t ( $C_{t}$ ); only the number of reported cases was assumed to be observed in the incidence time series dataset. As in the OG1 method, we assumed that the number of cases on day i of week t , $I_{7\left(t-1\right)+i}^{\left(7\right)}$ , was drawn from a Poisson distribution with mean given by equation (2.1). However, in the OG2 method, we additionally assumed that the number of reported cases on day i of week t , $C_{7\left(t-1\right)+i}^{\left(7\right)}$ , was then drawn from a binomial distribution with $I_{7\left(t-1\right)+i}^{\left(7\right)}$ trials and success probability ρ .

As in the OG1 method, we first estimated the value of $R_{2}$ , followed by $R_{3}$ and so on. To estimate $R_{2}$ , we ran simulations of the number of cases each day in week two (N.B. we simulated cases, rather than reported cases). In each simulation, we: (i) sampled $R_{2}$ from the prior (i.e. from a gamma distribution with shape parameter α and rate parameter β ); (ii) sampled the number of cases in week one based on the number of reported cases in the incidence dataset in week one (see electronic supplementary material, text S1); (iii) distributed the sampled number of cases in week one uniformly at random among the daily timesteps in week one; (iv) simulated the number of cases each day in week two. We repeated this procedure until M simulations had been generated in which the total number of simulated cases in week two exceeded the number of reported cases in the incidence dataset in week two.

This approach leads to a substantially higher proportion of simulations being ‘accepted’ than under the OG1 method, since any simulation in which the number of simulated cases in week two exceeds the number of reported cases in the dataset is included, allowing this method to be run quickly for large values of M . For each accepted simulation, we calculated the relative likelihood of that simulation given the observed number of cases in week two; this is simply the probability of observing the number of reported cases in the incidence dataset in week two from a binomial distribution in which the number of trials is the simulated number of cases in week two and the success probability is ρ (we refer to this relative likelihood quantity as the ‘weight’ of that simulation). For each accepted simulation, the number of cases each day (i.e. ${\left\{I_{i}^{\left(7\right)}\right\}}_{i=1}^{14}$ ) was stored, along with its corresponding weight. The mean estimate for $R_{2}$ was then determined as the weighted arithmetic mean of the values of $R_{2}$ in the accepted simulations. The central 95% credible interval for $R_{2}$ was also calculated accounting for the simulation weights.

To estimate $R_{t}$ for t≥3 weeks, we ran simulations of the number of cases each day in week t . In each simulation, we: (i) sampled $R_{t}$ from the prior (i.e. from a gamma distribution with shape parameter α and rate parameter β ); (ii) sampled the daily incidence of cases prior to week t from the matching sets stored when estimating $R_{t-1}$ (rather than sampling uniformly at random as in the OG1 method, the probability of sampling each set of daily incidence prior to week t was determined by the weights calculated when estimating $R_{t-1}$ ); (iii) simulated the number of cases each day in week t . We repeated this procedure until M simulations had been generated in which the total number of simulated cases in week t exceeded the number of reported cases in the incidence dataset in week t . For each accepted simulation, the number of cases each day (i.e. ${\left\{I_{i}^{\left(7\right)}\right\}}_{i=1}^{7t}$ ) was stored, along with its weight (the probability of observing the number of reported cases in the incidence dataset in week t from a binomial distribution in which the number of trials is the simulated number of cases in week t and the success probability is ρ ; this weight replaced the weight assigned when calculating $R_{t-1}$ ). The mean estimate for $R_{t}$ was again the weighted arithmetic mean of the values of $R_{t}$ underlying the accepted simulations. The 95% credible interval was again calculated accounting for the simulation weights.

**Figure 1.**
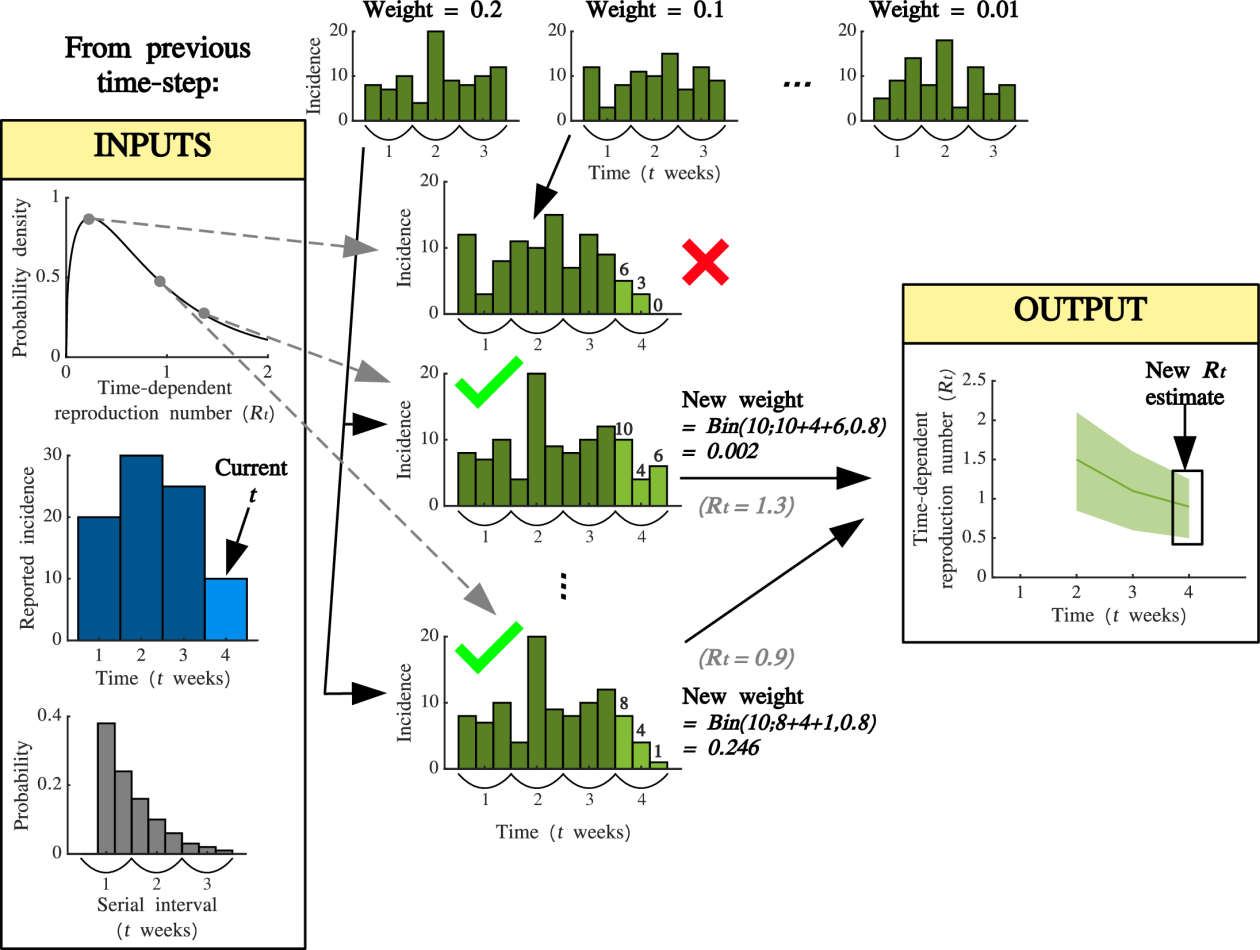
Schematic illustrating the steps taken to estimate **$R_{t}$** in any individual week **t** using the OG2 method. A value of $R_{t}$ is sampled from the prior, and the daily incidence of cases prior to week t is sampled from the matching sets stored when estimating $R_{t-1}$ (according to their weights). Daily incidence in week t is then simulated. If the number of cases simulated in week t exceeds the number of reported cases in that week in the incidence dataset, then that simulation is stored (green ticks) and its weight is calculated (the weight corresponds to the probability of observing the number of reported cases in week t given the simulated number of cases in that week, using a binomial distribution with reporting probability ρ ). These steps are then repeated until there are M simulations in which the number of simulated cases in week t exceeds the number of reported cases in that week. Those simulations are then used to construct the posterior estimate for $R_{t}$ (accounting for the weights).

A schematic illustrating the procedure for estimating $R_{t}$ using the OG2 method is provided in figure 1.

### (d) Serial interval

In all our analyses, we assumed that the serial interval distribution is a gamma distribution with mean 15.3 days and standard deviation 9.3 days. These Ebola-specific values were estimated in a previous study [32] from 178 empirical serial intervals recorded during the 2014 to 2016 epidemic in West Africa.

We discretized this distribution into daily ( $w^{\left(7\right)}$ ) or weekly ( w ) values using the approach outlined in the supplementary material of Ogi-Gittins *et al*. [20]. Specifically, denoting the probability density function of a gamma distribution with mean 2.19 weeks and standard deviation 1.33 weeks (these values correspond to 15.3 and 9.3 days, respectively) by g(x) , we calculated


$${w_{k}}^{\left(7\right)}=\int_{(k-1)/7}^{(k+1)/7}\left(1-7\left|u-\frac{k}{7}\right|\right)g(u)du,$$

for k=2,3,4… . This integral reflects the probability, based on the continuous serial interval distribution g(x) , that a specific infectee appears in the (discrete) disease incidence time series k days after their infector, under the assumption that the infector became a case at a time that is chosen uniformly at random within the relevant day. The value of ${w_{1}}^{\left(7\right)}$ was then chosen so that $w^{\left(7\right)}$ represents a valid probability distribution (i.e. its entries sum to one).

Similarly, we calculated


$$w_{k}=\int_{k-1}^{k+1}\left(1-\left|u-k\right|\right)g(u)du,$$

for k=2,3,4… and chose $w_{1}$ so that w is a valid probability distribution. Similarly to the above, this integral represents the probability that a specific infectee appears in the disease incidence time series k weeks after their infector, assuming that the infector became a case at a random time within the relevant week.

### (e) Outbreak datasets

#### (i) Simulated data

**Figure 2.**
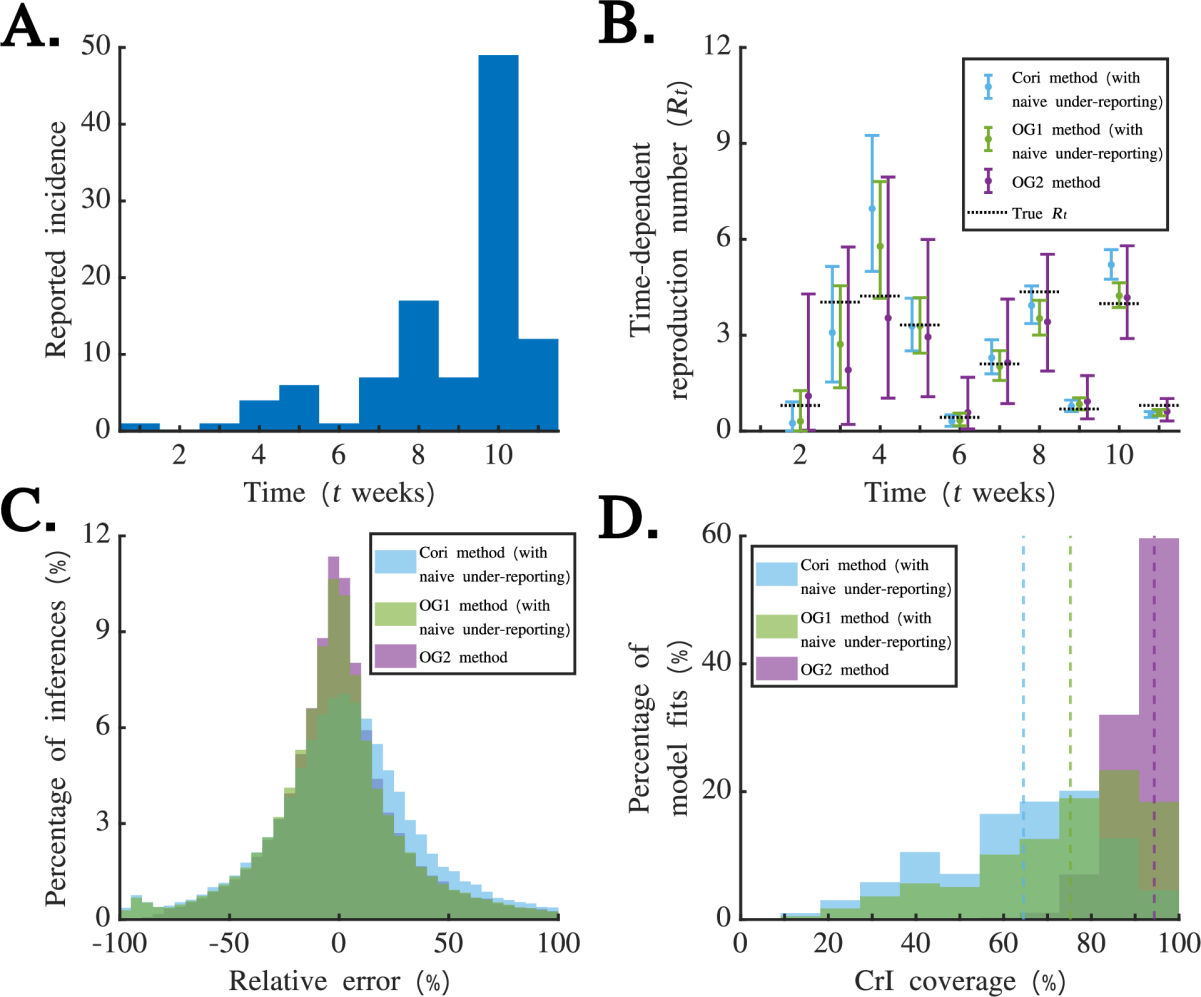
Comparing the performance of the OG2 method against application of the OG1 and Cori methods using simulated weekly disease incidence time series datasets. (A) A randomly chosen simulated dataset describing the number of reported cases each week generated under the assumption that ρ=0.1 (chosen from the small incidence datasets described in §2e(i)). (B) Estimates of $R_{t}$ obtained when the Cori method (blue), OG1 method (green) and OG2 method (purple) were applied to the dataset in panel (A). Dots represent mean estimates, error bars represent 95% credible intervals and the values of $R_{t}$ used to generate the dataset are also shown (black dotted). (C) The distribution of errors in mean estimates of $R_{t}$ each week across the 9000 simulated small incidence datasets when the Cori method (blue), OG1 method (green) and OG2 method (purple) were applied. These errors were calculated in each week by subtracting the true value of $R_{t}$ from the estimated value of $R_{t}$ and dividing by the true value of $R_{t}$ (and then converting the resulting values to percentages by multiplying by 100). (D) The distribution of credible interval coverage values across the 9000 simulated small incidence datasets when the Cori method (blue), OG1 method (green) and OG2 method (purple) were applied. The coverage for a single dataset represents the proportion of weeks in that dataset in which the true value of $R_{t}$ lies within the estimated 95% credible interval. Vertical dotted lines represent the mean coverage values across all datasets. In panels (B–D), we used a value of M=1000 when applying the OG1 method and a value of M=100000 when applying the OG2 method. When we applied the Cori method and the OG1 method, we first scaled the incidence data up by a multiplicative factor of 1/ρ to account for under-reporting (but neglecting stochasticity in reporting).

**Figure 3.**
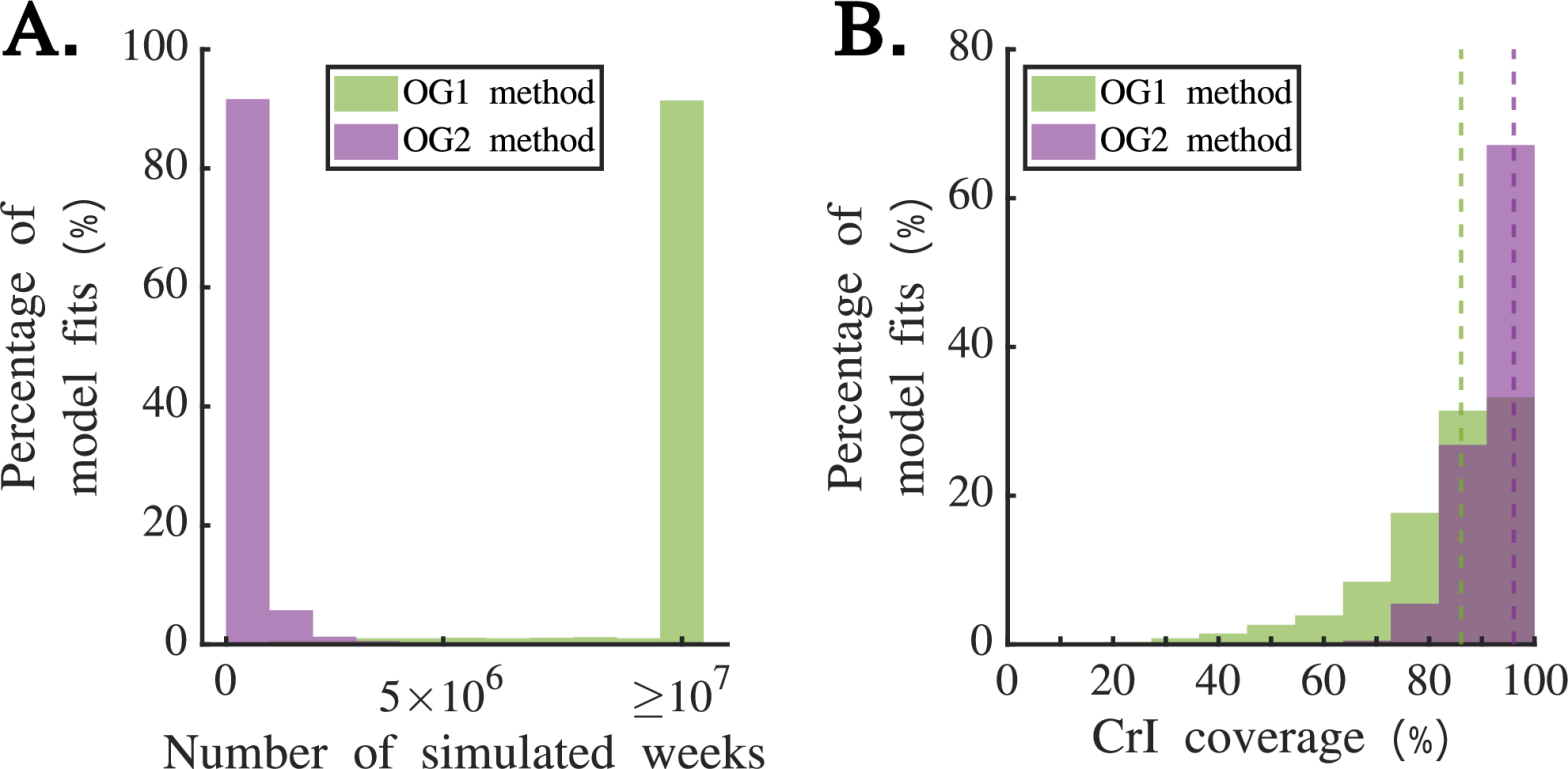
Comparing the computational efficiency of the OG1 and OG2 methods using simulated weekly disease incidence time series datasets. (A) The distribution of the number of weeks simulated as part of the inference procedure to obtain $R_{t}$ estimates when M=10000 , for the OG1 method (green) and the OG2 method (purple). After 10 million simulated weeks per dataset in the inference procedure, complete $R_{t}$ estimates had not yet been obtained for 91.4% of the large incidence datasets (§2e(i)) under the OG1 method (rightmost green bar). (B) The distribution of credible interval coverage values between simulated datasets (i.e. the proportion of timepoints in each dataset for which the true values of $R_{t}$ used to generate the dataset lay within the 95% credible interval obtained in the inference procedure) for the OG1 method (green) and OG2 method (purple) after similar computation times. Dotted vertical lines reflect the mean coverage values under each method. In panel (B), to obtain comparable computation times for the two methods, we set M=10000 when we used the OG2 method, and then chose M (separately for each dataset) when we used the OG1 method so that the number of simulated weeks in the inference procedure was comparable but higher for the OG1 method than for the OG2 method. For a randomly chosen dataset, we repeated our inference using the OG2 method 30 times for different values of M , finding that a value of M=10000 was sufficient to obtain a consistent mean estimate of $R_{t}$ and consistent boundaries of the 95% credible interval for $R_{t}$ (electronic supplementary material, fig. S1). In the analysis shown in this figure, we applied the OG1 method directly to the simulated reported incidence datasets (without first scaling the datasets to reflect under-reporting).

Before applying the OG2 method to real-world data, we first tested its performance against the Cori method and the OG1 method using simulated datasets. In §3a (figure 2), we consider 9000 simulated datasets with relatively small numbers of cases to investigate the effect of stochasticity in reporting on $R_{t}$ inference. In §3b (figure 3 and electronic supplementary material, figs. S1 and S2), we consider 9000 simulated datasets with large numbers of cases to compare the computational resources required to run the OG1 and OG2 methods. Throughout this article, we refer to the first set of datasets as ‘small incidence’ datasets, and the second set of datasets as ‘large incidence’ datasets.

We first describe the procedure that we used to generate the small incidence datasets (analysed in §3a). We initially generated 1000 simulated outbreaks using a renewal equation with a daily timestep in which the number of cases each day was drawn from a Poisson distribution with mean given by equation (2.1). Each simulation was initiated with a single case on the first day, and simulations were run for 11 weeks. In each simulation, $R_{t}$ was assumed to change each week, with each value of $R_{t}$ sampled independently from a gamma distribution with shape parameter k=1 and rate parameter γ=1/2 (so that the gamma distribution had mean value k/γ=2 ). This process generated 1000 simulated sets of daily incidence of cases, which we aggregated into weekly values. We then considered nine different values of the reporting probability, ρ=0.1,0.2,…,0.9 . Starting with ρ=0.1 , for each of the 1000 sets of weekly incidence we sampled the number of reported cases each week, assuming that the number of reported cases is drawn from a binomial distribution (with the number of trials set to be the incidence of cases for that week and the success probability set to be ρ=0.1 ). We then repeated this process for all nine values of ρ , generating 9000 synthetic datasets in total describing the weekly incidence of reported cases (1000 datasets for each value of ρ) .

To instead generate the large incidence datasets (analysed in §3b), we followed the same procedure but instead drew each value of $R_{t}$ independently from a gamma distribution with shape parameter k=1 and rate parameter γ=1/3 (so that the gamma distribution had mean value k/γ=3 ).

#### (ii) Ebola outbreak in the DRC, 2018−2020

We conducted analyses using real-world data from the 2018 to 2020 Ebola outbreak in the North Kivu, Ituri and South Kivu provinces of the DRC [33]. In that outbreak, 3470 cases were reported (3317 confirmed cases and 153 probable cases). A disease incidence time series describing the number of cases each week was available for cases with recorded symptom onset dates (3438 cases), and we used that incidence time series in our analyses.

## 3. Results

### (a) Comparison of *R*_*t*_ estimates from the Cori, OG1 and OG2 methods

We began by comparing the performance of the Cori, OG1 and OG2 methods using simulated data. In figure 2A, the incidence of reported cases is shown for a simulated incidence dataset (randomly chosen from the 1000 small incidence datasets in which the case reporting probability is ρ=0.1 ; see §2e(i)). Corresponding estimates of $R_{t}$ are shown in figure 2B. The Cori method does not account for weekly aggregation of the disease incidence data or under-reporting. The OG1 method accounts for weekly aggregation of the disease incidence data but does not account for under-reporting. To partially account for under-reporting, when we applied the Cori and OG1 methods in figure 2, we first scaled the incidence data by a constant multiplicative factor of 1/ρ (and rounded up any non-integer case numbers); consequently, our application of those methods accounted for under-reporting, but not in a stochastic fashion. The OG2 method accounts for both weekly aggregation of the disease incidence data and stochastic under-reporting. Notably, in figure 2B, there are timesteps in which the true value of $R_{t}$ lies outside the credible intervals obtained from the Cori method ( t=4 weeks, t=10 weeks and t=11 weeks) and the OG1 method ( t=8 weeks and t=11 weeks). This indicates that the OG2 method may provide more reliable estimates of $R_{t}$ than either the Cori method or the OG1 method when the disease incidence dataset is subject to temporal aggregation and under-reporting.

We then tested whether this conclusion holds across the 9000 simulated small incidence datasets by comparing the error in the mean estimates of $R_{t}$ each week (compared to the true underlying values of $R_{t}$ ) obtained from each method (figure 2C). In this analysis, a positive error value corresponds to an instance in which the estimated $R_{t}$ value is higher than the true value (and vice versa for a negative error value). In general, the OG1 and OG2 methods provided more accurate mean estimates of $R_{t}$ than the Cori method. Of course, despite accounting for both weekly aggregation of the disease incidence data and under-reporting, the mean estimates from the OG2 method did not equal the true values of $R_{t}$ exactly owing to stochasticity in the weekly case numbers, particularly early in the simulated outbreaks when case numbers were particularly low. The mean value of the absolute relative error was 26% for the OG2 method, compared to 31% for the Cori method and 27% for the OG1 method. We also checked the credible interval coverage for each simulated dataset (i.e. the proportion of estimated $R_{t}$ values in each dataset lying within the 95% credible interval; figure 2D). We found that 94% of $R_{t}$ values inferred using the OG2 method lay within the credible interval (appropriately, and as expected, the coverage from the OG2 method was very close to 95%), compared to 64% using the Cori method and 75% using the OG1 method.

The results shown in figure 2C,D provide insights into the factors underlying differences in the inferred $R_{t}$ values between the methods. In figure 2C, similar error values from the OG1 and OG2 methods indicate that erroneous mean $R_{t}$ estimates from the Cori method were in part due to temporal aggregation of the disease incidence time series. However, the credible interval coverage differed between the OG1 and OG2 methods (figure 2D), suggesting that stochastic under-reporting affects credible intervals in $R_{t}$ estimates. Accounting for under-reporting in a stochastic fashion therefore generates appropriate uncertainty in the disease incidence time series, with implications for uncertainty in $R_{t}$ estimates.

### (b) Computational efficiency of the OG1 and OG2 methods

Having demonstrated that the OG2 method generates robust $R_{t}$ estimates in the presence of weekly aggregation of disease incidence data and under-reporting, we went on to test the computational efficiency of the OG1 method (which accounts for temporal aggregation of the incidence data but does not attempt to account for under-reporting) and the OG2 method. We undertook this analysis because implementing the OG1 method was the most computationally intensive aspect of generating the results shown in figure 2.

We applied the OG1 and OG2 methods to the 9000 simulated large incidence datasets described in §2e(i) (figure 3; we analysed the large incidence datasets here to consider a scenario in which $R_{t}$ inference using simulation-based approaches might be expected to require substantial computational resources). We found that, for a fixed value of M=10000 , the OG2 method allowed $R_{t}$ to be inferred quickly, with $R_{t}$ inference being completed in 99.7% of the simulated datasets by the time that 10 million weeks (for each dataset) had been simulated in the inference procedure (purple bars in figure 3A). In contrast, after 10 million simulated weeks in the inference procedure for each dataset, only 8.6% of simulated datasets had complete $R_{t}$ estimates using the OG1 method for the same fixed value of M=10000 (green bars in figure 3A). We note that, in figure 3, when we applied the OG1 method we did not scale up the incidence data to account for under-reporting. Had we done so, $R_{t}$ inference using the OG1 method would have been even more time consuming, further highlighting the computational benefits of using the OG2 method rather than the OG1 method.

We then contrasted the quality of $R_{t}$ estimates obtained from the simulated large incidence datasets using the OG1 and OG2 methods in comparable computation times (figure 3B). Specifically, we fixed M=10000 in the OG2 method, and then chose M when we applied the OG1 method to each simulated dataset so that the total numbers of simulated weeks in the inference procedure were similar when both methods were applied. To obtain a conservative estimate of the extent to which inference was improved under the OG2 method compared to the OG1 method, when we used the OG1 method we chose the smallest value of M (for each dataset separately) for which the number of simulated weeks in the inference procedure was larger for the OG1 method. The mean value of M used across all 9000 simulated large incidence datasets when the OG1 method was applied was then M=78 (compared to the fixed value of M=10000 under the OG2 method). We found that, under the OG1 method, only 86% of inferred $R_{t}$ values lay within the 95% credible interval (as opposed to 96% under the OG2 method). We note that, aside from the different simulated datasets considered, the key difference between the results shown for the OG1 method in figures 2D and 3B is that, in figure 2D, we used a fixed value of M=1000 when applying the OG1 method, whereas in figure 3B we chose the value of M when applying the OG1 method to ensure similar computation times compared to when we used the OG2 method.

**Figure 4.**
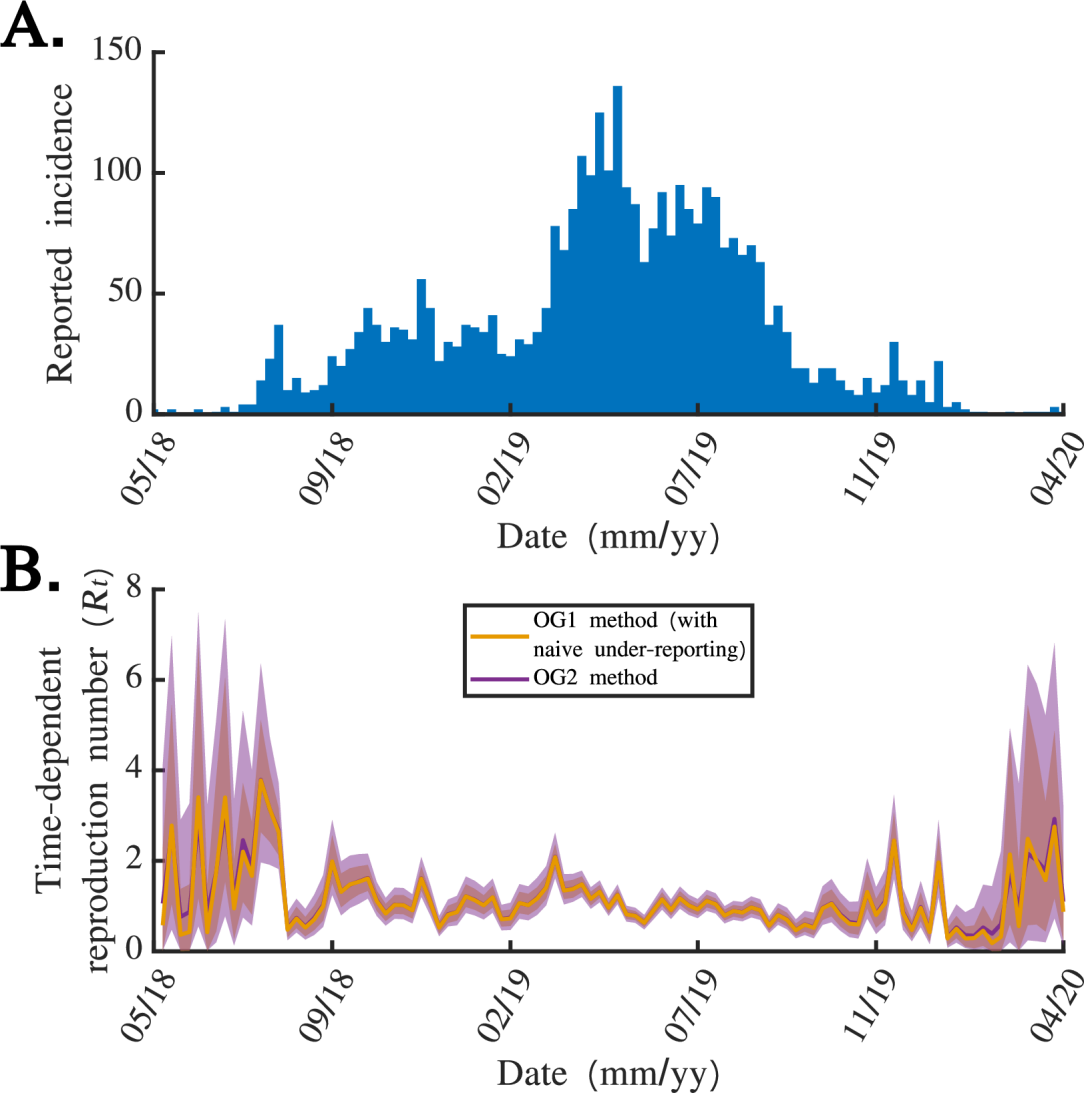
The effect of stochasticity in reporting on $R_{t}$ estimates, using data from the 2018 to 2020 Ebola outbreak in the DRC. (A) Number of reported cases each week in the 2018 to 2020 Ebola outbreak in the DRC. (B) Application of the OG1 method to the disease incidence time series in panel (A) after first multiplying the number of reported cases each week by 1/ρ=2.5 (yellow), and application of the OG2 method (with ρ=0.4 ) directly to the incidence time series in panel *(*A) (purple). Solid lines represent mean estimates and shaded areas represent 95% credible intervals. We used a value of M=1000 when applying the OG1 method and a value of M=100000 when applying the OG2 method.

We went on to apply the OG1 and OG2 methods to the incidence dataset from the 2018 to 2020 Ebola outbreak in the DRC (figure 4A). To investigate the effect of accounting for stochasticity in reporting on $R_{t}$ estimates in that real-world setting, we compared results from the OG2 method (which accounts for stochastic reporting) to analogous results from the OG1 method applied to a disease incidence time series that has been scaled up to account for under-reporting in a deterministic fashion. Specifically, we assumed that ρ=0.4 when we applied the OG2 method (consistent with the under-reporting correction factor used by the United States Centers for Disease Control early in the 2014 to 2016 Ebola epidemic [34]), whereas when we applied the OG1 method we first multiplied the number of reported cases each day by 1/ρ=2.5 (then rounding up any non-integer case numbers). We found that failing to account for stochasticity in reporting led to narrower credible intervals in $R_{t}$ estimates (cf. yellow and purple credible intervals in figure 4B). This is because failing to account for stochasticity in reporting implicitly provides too much confidence in the true number of cases each week, which translates into inappropriately high certainty in $R_{t}$ estimates.

### (c) The effect of the extent of under-reporting on *R*_*t*_ inference

**Figure 5.**
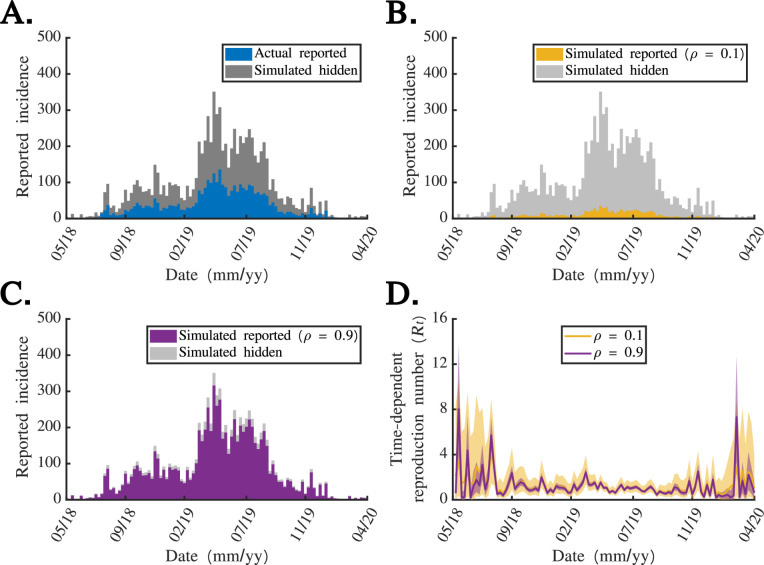
Effect of the reporting probability on the precision of $R_{t}$ estimates, using data from the 2018 to 2020 Ebola outbreak in the DRC. (A) Number of reported cases each week in the 2018 to 2020 Ebola outbreak in the DRC (blue), and the underlying ‘true’ number of cases each week (grey) obtained by sampling under the assumption that the number of reported cases each week is drawn from a binomial distribution with ρ=0.4 . The height of each grey bar therefore represents the simulated total number of cases (including both reported and unreported cases) in any given week. (B) ‘True’ number of cases each week (grey; identical to grey bars in panel (A)) and the number of reported cases each week under the assumption that ρ=0.1 (yellow; obtained by multiplying the ‘true’ number of cases each week by ρ and then rounding non-integer values up). (C) ‘True’ number of cases each week (grey; identical to grey bars in panel (A)) and the number of reported cases each week under the assumption that ρ=0.9 (purple; obtained by multiplying the ‘true’ number of cases each week by ρ and then rounding non-integer values up). (D) Estimates of $R_{t}$ obtained from the OG2 method from the reported cases data shown in panels (B) (yellow) and (C) (purple). Solid lines represent mean estimates and shaded areas represent 95% credible intervals. In both scenarios, we used a value of M=100000 .

We conducted an analysis into the effect of the level of under-reporting on $R_{t}$ estimates obtained using the OG2 method. To do this, we first considered the reported weekly disease incidence from the 2018 to 2020 Ebola outbreak in the DRC (figure 4A) and sampled the ‘true’ underlying case numbers under the assumption that the number of reported cases each week was drawn from a binomial distribution with reporting probability ρ=0.4 (figure 5A). This provided us with a ‘dataset’ describing true weekly case numbers from which to test the effects of different levels of under-reporting.

We then considered two counterfactual scenarios. In the first, we considered a scenario with a low reporting probability ( ρ=0.1 ; figure 5B). In the second, we considered a scenario with a high reporting probability ( ρ=0.9 ; figure 5C). In each scenario, we generated synthetic reported cases data by multiplying the number of cases each week by ρ , leading to the numbers of reported cases shown in yellow and purple in figure 5B,C. We then used those data to infer $R_{t}$ using the OG2 method (figure 5D). We found that, under the assumption that the reporting probability is low, there was substantially more uncertainty in the estimated $R_{t}$ values. In particular, in figure 5D, the 95% credible interval when ρ=0.1 was 3.46 times as wide each week (on average) compared to the credible interval when ρ=0.9 . This is because, when the OG2 method is applied, reporting is assumed to be a stochastic process. A low value of ρ then corresponds to more uncertainty in the disease incidence time series, which carries over into uncertainty in $R_{t}$ . This highlights that the implementation of surveillance strategies to increase ρ has the potential to increase the certainty in $R_{t}$ estimates.

We obtained similar conclusions when we instead analysed the simulated large incidence datasets described in §2e(i), finding that both errors in central estimates of $R_{t}$ and the width of the 95% credible interval decreased with increasing values of ρ (electronic supplementary material, fig. S2).

### (d) Extending the OG2 method to account for temporal variations in reporting

In most of our analyses using the OG2 method, we assumed that the case reporting probability, ρ , remains constant throughout the outbreak. To demonstrate the straightforward extensibility of the OG2 method, we also conducted additional analyses in which the reporting probability was assumed to vary temporally. When applying the OG2 method, this simply involved replacing ρ with the relevant value of $\rho_{t}$ when running simulations and calculating the simulation weights. Further details about these analyses are provided in electronic supplementary material, text S2.

**Figure 6.**
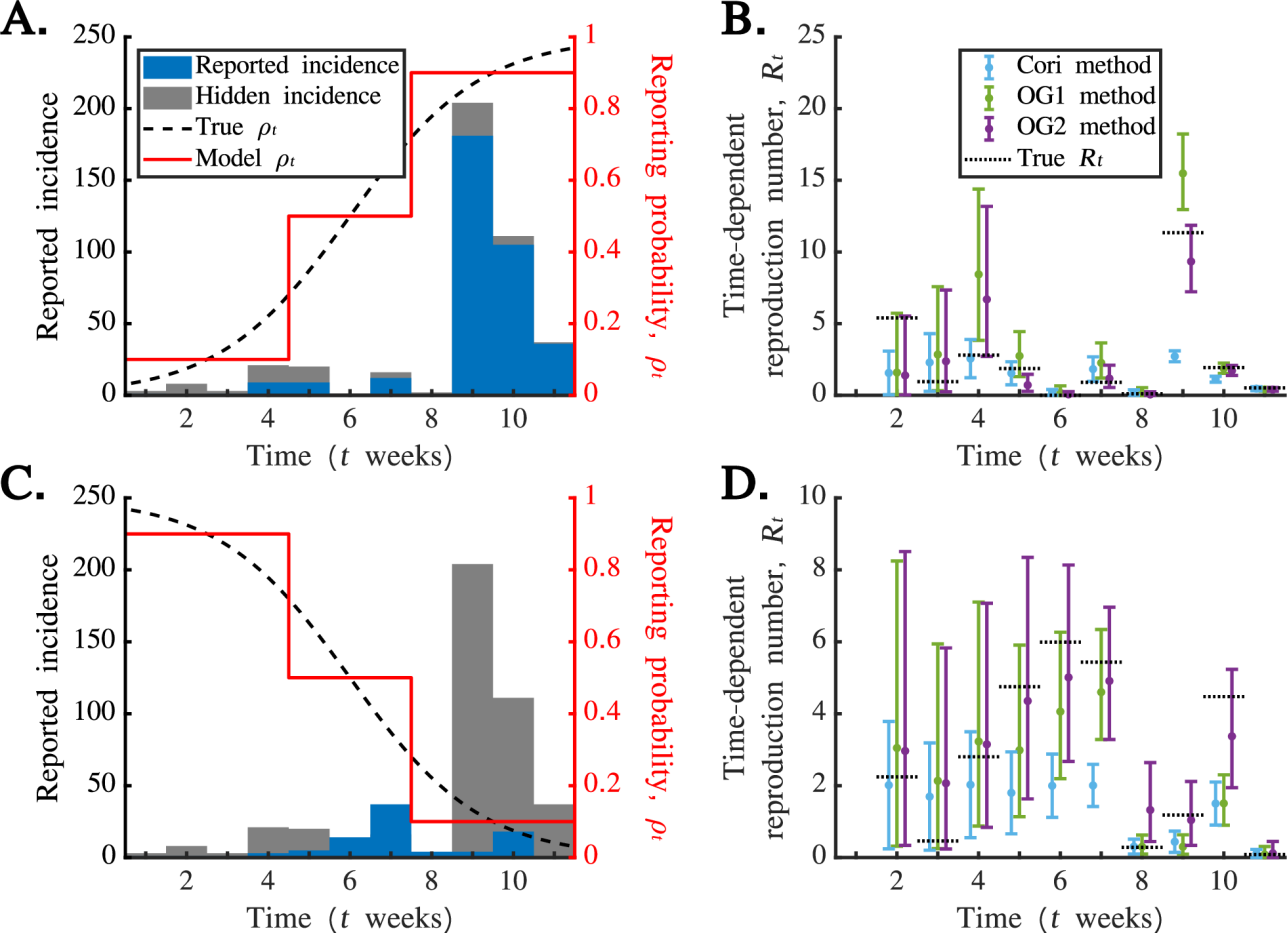
Comparing the performance of the OG2 method against application of the OG1 and Cori methods when the level of case reporting varies temporally. (A) A simulated dataset describing the number of reported cases each week generated under the assumption that case reporting improves during the outbreak (blue bars; cases that are not reported are indicated by the grey bars). The case reporting probability used to generate the data is shown (black dotted), alongside the case reporting probability that was used when inferring $R_{t}$ using the OG2 method in panel (B) (red). (B) Estimates of $R_{t}$ obtained when applying the Cori method (blue), OG1 method (green) and OG2 method (purple) to the dataset in panel (A). Dots represent mean estimates, error bars represent 95% credible intervals and the values of $R_{t}$ used to generate the dataset are also shown (black dotted). (C) Analogous to panel (A), but for a simulated outbreak dataset in which case reporting worsens during the outbreak. (D) Analogous to panel (B), but showing $R_{t}$ estimates obtained from the dataset in panel (C). In panels (B) and (D), we used a value of M=1000 when applying the OG1 method and a value of M=100000 when applying the OG2 method. In the analysis shown in this figure, we applied the Cori and OG1 methods directly to the simulated reported incidence datasets (without first scaling the datasets to account for under-reporting).

In figure 6A, we present a simulated disease incidence time series dataset generated under the assumption that $\rho_{t}$ increases throughout the outbreak (black dotted line in figure 6A). Corresponding estimates of $R_{t}$ are shown in figure 6B. The Cori and OG1 methods do not account for under-reporting, whereas the OG2 method does. Since precise temporal changes in $\rho_{t}$ would be unlikely to be known in practice, when applying the OG2 method we assumed that a step function approximation of $\rho_{t}$ was known (red line in figure 6A), as might be obtained if the reporting probability was estimated in separate analyses undertaken at different outbreak stages (see further discussion in §4). We then repeated this analysis for two other simulated disease incidence time series datasets in which $\rho_{t}$ was assumed to increase during the outbreaks (electronic supplementary material, fig. S3A–D), and for three simulated disease incidence time series datasets in which $\rho_{t}$ was assumed to decrease during the outbreaks (figure 6C,D and electronic supplementary material, fig. S3E–G). In all six simulated outbreaks considered, we found that the OG2 method provided more robust estimates of $R_{t}$ than the Cori or OG1 methods; the true value of $R_{t}$ was most likely to lie in the inferred 95% credible interval using the OG2 method.

## 4. Discussion

Evaluating temporal changes in pathogen transmission during infectious disease outbreaks is an important challenge for epidemiological modellers. The time-dependent reproduction number, $R_{t}$ , is an intuitive metric that can be estimated and tracked during outbreaks. However, the reliability of $R_{t}$ inference is negatively affected by key features of real-world outbreaks, including inaccurate case reporting and temporal aggregation of disease incidence time series data.

In this article, we have built on our earlier method for estimating $R_{t}$ [20] (referred to here as the OG1 method), which was designed for use in scenarios in which disease incidence time series data are aggregated temporally. While the OG1 method provides a framework for inferring $R_{t}$ that is straightforward to apply, two main issues are associated with it. First, the method can require substantial computational resource to run, since it involves repeatedly simulating a stochastic epidemiological model until a large number of simulations have been generated that match the observed disease incidence time series dataset exactly. This may be slow for large incidence datasets, even though the OG1 method uses an iterative simulation approach to improve computational efficiency. Second, the OG1 method does not account for stochastic case under-reporting, with implications for the precision of $R_{t}$ estimates. While the speed of the OG1 method could be increased by relaxing the requirement for simulations to match the real-world dataset exactly (analogously to the use of tolerance values in Approximate Bayesian Computation [35,36]), this would come at the cost of lower accuracy in $R_{t}$ estimates, and under-reporting would still not be accounted for.

We therefore introduced the OG2 method, which enables $R_{t}$ estimates to be generated more quickly than using the OG1 method and accounts for stochastic under-reporting in a rigorous fashion. The computational efficiency of the OG2 method stems from the fact that information about the value of $R_{t}$ is obtained from any simulation in which the number of simulated cases in a given week exceeds the number of reported cases, thereby removing the limitation that most model simulations are ‘thrown away’ under the OG1 method. We first used 9000 simulated datasets (the small incidence datasets described in §2e(i)) to demonstrate that the OG2 method leads to more accurate and more precise estimates of $R_{t}$ than naive application of the widely used Cori method to under-reported and weekly aggregated disease incidence time series, even if the incidence data are scaled up to reflect under-reporting when applying the Cori method (figure 2). We also showed that, in the presence of stochastic under-reporting, the OG2 method generates $R_{t}$ estimates in which uncertainty is reflected more appropriately than under the OG1 method; when the OG2 method was applied, the true values of $R_{t}$ lay within the credible intervals approximately 95% of the time across the simulated datasets (figure 2D). We then compared the performance of the OG1 and OG2 methods using other simulated datasets (the large incidence datasets described in §2e(i)) to demonstrate the superior computational efficiency of the OG2 method (figure 3A) and the improved performance of the OG2 method compared to OG1 method when there are restrictions in available computational resources (figure 3B). We then went on to apply the OG2 method to data from the 2018 to 2020 Ebola outbreak in the DRC, again showing that failing to account for stochasticity in reporting leads to inappropriately precise $R_{t}$ estimates (figure 4) and that the level of under-reporting affects inferred credible intervals in $R_{t}$ estimates substantially (figure 5). Finally, we applied the Cori, OG1 and OG2 methods to simulated datasets in which the reporting probability was assumed to change during the outbreaks (figure 6 and electronic supplementary material, fig. S3). We found that even imperfect knowledge of variations in reporting can provide useful information when estimating $R_{t}$ , and demonstrated how such information can be accounted for when applying the OG2 method.

The research presented in this article builds on a substantial number of previous publications about $R_{t}$ inference in the literature, which includes some methods for estimating $R_{t}$ in the presence of temporal aggregation of disease incidence time series or under-reporting. As described in §1, earlier methods for estimating $R_{t}$ by Nash *et al.* [21] and Ogi-Gittins *et al.* [20] have accounted for temporal aggregation of disease incidence data, but did not consider uncertainty in $R_{t}$ estimates generated by under-reporting. An example of a study in which $R_{t}$ estimation has been undertaken while accounting for under-reporting is the article by Hong & Li [37], in which $R_{t}$ was inferred based on a discrete time Susceptible-Infected-Removed model from data describing the daily incidence of reported cases. Dalziel *et al.* [23] developed a framework for estimating $R_{t}$ for Ebola from weekly burial records while accounting for under-reporting. In that study, the Cori method was applied to estimated daily incidence data.

As with any epidemiological modelling study, our analyses were based on simplifying assumptions. Perhaps the main limiting assumption of our analyses is that the probability that a case is reported, ρ , was assumed to be known when estimating $R_{t}$ (although known imperfectly in figure 6 and electronic supplementary material, fig. S3). For our method to be applied in practice, it would therefore be necessary to assume or estimate the value of ρ , potentially considering different values of ρ at different stages of the outbreak (as in figure 6 and electronic supplementary material, fig. S3). One possible approach for doing this could be to undertake rigorous testing for infection (or a serosurvey) in a specific localized region, comparing the resulting number of detected cases against the number of cases identified through routine notification routes [25]. Consequent estimates of ρ could then be assumed across the entire population. Alternative methods have been proposed for estimating reporting probabilities in epidemiological models, including using alternative data sources in conjunction with data on reported cases (e.g. data describing numbers of deaths or hospitalizations [38], or wastewater data [39]). A possible extension to the OG2 method is to account for uncertainty in the value of ρ , for example by using a distributional estimate of ρ rather than a fixed value. Another potential extension is to account for heterogeneity in the reporting probability between different groups within the population [40], accounting for disparities between ethnic groups or factors such as different levels of symptoms in individuals of different ages [41,42].

In addition, as an extension to the OG1 method, the OG2 method inherits many of the original assumptions of the OG1 method. For example, when we applied the OG1 and OG2 methods, we made the common assumption that the number of cases on any day was drawn from a Poisson distribution. In principle, this assumption could be altered, with one possibility being to instead use a negative binomial distribution to characterize the number of cases each day (this would allow for the possibility of super-spreading events occurring on some days, if the dispersion parameter of the negative binomial distribution is chosen appropriately [43,44]). Another avenue for further research is to explore whether inferring $R_{t}$ from weekly aggregated data using the OG2 method may sometimes be preferable to inferring $R_{t}$ from daily disease incidence data, even if daily data are available. For example, if the extent of under-reporting varied during the week in a complex fashion (e.g. owing to a day-of-the-week effect [45,46]), then more robust estimates might be obtained from weekly aggregated, rather than daily, incidence data.

In conclusion, we have presented a novel simulation-based method—the OG2 method—for inferring $R_{t}$ . The OG2 method performs favourably compared to previous approaches when reported disease incidence time series data are subject to both temporal aggregation (e.g. weekly reporting) and under-reporting. Since these features affect surveillance for a wide range of diseases, we hope that the methodological advance presented here will be useful for estimating $R_{t}$ during future infectious disease outbreaks.

## Data Availability

The computing code used to perform the analyses in this article is available in the following GitHub repository: https://github.com/billigitt/UnderreportedAndTemporallyAggregated. Code was written in MATLAB (compatible with v. 2021b) and Julia (compatible with v. 1.11.1). Supplementary material is available online [47].
